# Generation and Analysis of a Large-Scale Expressed Sequence Tag Database from a Full-Length Enriched cDNA Library of Developing Leaves of *Gossypium hirsutum* L

**DOI:** 10.1371/journal.pone.0076443

**Published:** 2013-10-11

**Authors:** Min Lin, Deyong Lai, Chaoyou Pang, Shuli Fan, Meizhen Song, Shuxun Yu

**Affiliations:** State Key Laboratory of Cotton Biology, Institute of Cotton Research of CAAS, Anyang, Henan, P. R. China; New Mexico State University, United States of America

## Abstract

**Background:**

Cotton (*Gossypium hirsutum* L.) is one of the world’s most economically-important crops. However, its entire genome has not been sequenced, and limited resources are available in GenBank for understanding the molecular mechanisms underlying leaf development and senescence.

**Methodology/Principal Findings:**

In this study, 9,874 high-quality ESTs were generated from a normalized, full-length cDNA library derived from pooled RNA isolated from throughout leaf development during the plant blooming stage. After clustering and assembly of these ESTs, 5,191 unique sequences, representative 1,652 contigs and 3,539 singletons, were obtained. The average unique sequence length was 682 bp. Annotation of these unique sequences revealed that 84.4% showed significant homology to sequences in the NCBI non-redundant protein database, and 57.3% had significant hits to known proteins in the Swiss-Prot database. Comparative analysis indicated that our library added 2,400 ESTs and 991 unique sequences to those known for cotton. The unigenes were functionally characterized by gene ontology annotation. We identified 1,339 and 200 unigenes as potential leaf senescence-related genes and transcription factors, respectively. Moreover, nine genes related to leaf senescence and eleven MYB transcription factors were randomly selected for quantitative real-time PCR (qRT-PCR), which revealed that these genes were regulated differentially during senescence. The qRT-PCR for three *GhYLSs* revealed that these genes express express preferentially in senescent leaves.

**Conclusions/Significance:**

These EST resources will provide valuable sequence information for gene expression profiling analyses and functional genomics studies to elucidate their roles, as well as for studying the mechanisms of leaf development and senescence in cotton and discovering candidate genes related to important agronomic traits of cotton. These data will also facilitate future whole-genome sequence assembly and annotation in *G. hirsutum* and comparative genomics among *Gossypium* species.

## Introduction

Cotton (*Gossypium* spp.) is the world’s most important agronomic fiber, as well as a significant oilseed crop. The seed is an important source of feed, foodstuff, and oil. The crop is widely cultivated in more than 80 countries, with China, India, the United States of America, and Pakistan the top four cotton producers (http://www.cotton.org/econ/cropinfo/cropdata/rankings.cfm). China is the largest producer and consumer of raw cotton. *Gossypium hirsutum* L., or upland cotton, is a primary cultivated species and has an allotetraploid genome (AD; 2n = 4x = 52). *Gossypium hirsutum* produces over 90% of the world’s fibers because of its higher yield and wider environmental adaptability [1], [2].

The advent of new molecular genetic technologies and the dramatic increase in plant gene sequence data have provided opportunities to understand the molecular basis of traits important for plant breeding, such as improved yield and plant quality. The entire genomic sequence is not available for *G. hirsutum*, but a large number of genomic resources have been developed for this species. These include bacterial artificial chromosomes (BACs) [3], polymorphic markers [4], and genome-wide cDNA-based or unigene expressed sequence tag (EST)–based microarrays [5]. A rapid and cost-efficient way to acquire transcriptome data for an organism with a large, complex, and unknown genome is EST sequencing; analysis of ESTs can also complement whole-genome sequencing [6]. ESTs are short, single-pass sequence reads from mRNA (cDNA). Large scale EST data represent a snapshot of genes expressed in a given tissue and/or at a given developmental stage. They are tags of expression for a given cDNA library [7]. Large EST datasets can be used to discover novel genes and carry out functional genetic studies [8], [9]. So far, a large number of *G.* *hirsutum* ESTs have been produced from cDNA libraries constructed from fibers, ovules, bolls, roots, and stems. The overwhelming majority of these EST resources have focused on fiber-development organs and have been used to explore the key genes involved in fiber development and its mechanism [10], [11]. However, large-scale EST data related to leaf development are lacking.

Leaves are specialized photosynthetic organs, and plants harvest energy and nutrients in their production. Leaf development encompasses many distinct stages, from leaf primordium formation to expansion, maturation, and abscission. The onset and progression of leaf senescence, the last phase, is accompanied by changes in expression of a large number of senescence-associated genes (SAGs). Some genes must be newly activated in leaves for the onset of senescence [12], [13]. Premature senescence, when the plant drops its leaves too early, has been occurring at an increasing frequency since the introduction of modern, high-yielding cotton cultivars like *Bacillus thuringiensis* (Bt) cotton. Premature leaf senescence results in reduced lint yield and poor fiber properties in cotton [14]. Understanding the molecular mechanisms of leaf senescence could greatly enhance yield and quality by guiding appropriate management to avoid premature leaf loss.

In recent decades, many advances in the understanding of leaf senescence at the molecular level have been achieved in several species, such as *Arabidopsis thaliana* and rice, by different experimental methods. Nine yellow-leaf-specific genes (*YLS*) were isolated, and RNA gel blot analysis revealed that most of them were senescence-up-regulated; the expression characteristics of *YLS* genes will be useful as potential molecular markers [15]. Transcript abundance in leaves of *Populus tremula* was studied by microarrays obtained from seven cDNA libraries, and 677 significantly up-regulated genes were identified during leaf senescence. The evidence for increased transcriptional activity before the appearance of visible signs of senescence was also found [16]. In *Medicago truncatula* leaves, 545 differentially-expressed genes, including 346 senescence-enhanced and 199 repressed genes, were identified by cDNA amplified fragment length polymorphism (AFLP) techniques; comparison with *Arabidopsis* datasets revealed common physiological events but differences in nitrogen metabolism and transcriptional regulation [17]. In rice, 533 differentially expressed genes were isolated by suppression subtractive hybridization (SSH) from early-senescent flag leaves, 183 had gene ontology (GO) annotations indicating involvement in macromolecule metabolism, protein biosynthesis regulation, energy metabolism, detoxification, pathogenicity and stress, and cytoskeleton organization [18]. A total of 140 annotated up-regulated genes in wheat flag leaves were analyzed using an in-house fabricated cDNA microarray. The results supported a protective role of mitochondria towards oxidative cell damage via the up-regulation of an alternative oxidase and possibly also succinate dehydrogenase [19]. During natural leaf senescence in *Arabidopsis*, 827 SAGs were identified. Comparison of these genes with artificially-induced senescence suggested that alternative pathways for essential metabolic processes such as nitrogen mobilization were used in different senescent systems [20]. Recently, a high-resolution time-course profile of gene expression during leaf senescence was obtained by microarray analysis. The dynamic changes in transcript levels were identified globally as senescence progresses, and the involvement of metabolic processes, signaling pathways, and specific transcription factors (TFs) were explicitly clarified [21]. Among the SAGs, many TFs, receptors, signaling components for hormones and stress responses, and regulators of metabolism were involved in regulating leaf senescence, indicating that senescence is governed by complex transcriptional regulatory networks.

In this study, a normalized and full-length cDNA library from different developmental stages of *G. hirsutum* leaves was constructed. Random sequencing of clones from the cDNA library generated a total of 9,874 high-quality ESTs, which were assembled into 5,191 unique sequences, consisting of 1,652 contigs and 3,539 singletons. Several SAGs and TFs were identified. This work will benefit the study of leaf senescence mechanisms of *G.* *hirsutum*, form a foundation for cloning the full-length sequences of these genes for genetic engineering, and also provide important resources for comparative genomic studies among closely-related species.

## Results

### Characterization of cDNA Library and EST Sequencing and Assembly

A normalized full-length cDNA library was constructed using leaves during the plant flowering stage. To evaluate the fullness ratios of the cDNA inserts of the library, 50 clones were randomly selected and fully sequenced; 44 (88%) contained putative full-length sequences. To assess the normalization efficiency, the relative concentration of 18S ribosomal RNA (18S) and actin in both the non-normalized and normalized cDNA populations were estimated by quantitative real-time PCR (qRT-PCR). The differences in cycle number (ΔCt) increased by 7.18 and 7.65 after library normalization, respectively. The results showed that the copies of these two genes decreased 145 and 200 fold, respectively, and suggested that the normalization quality of this library was good.

**Figure 1:**
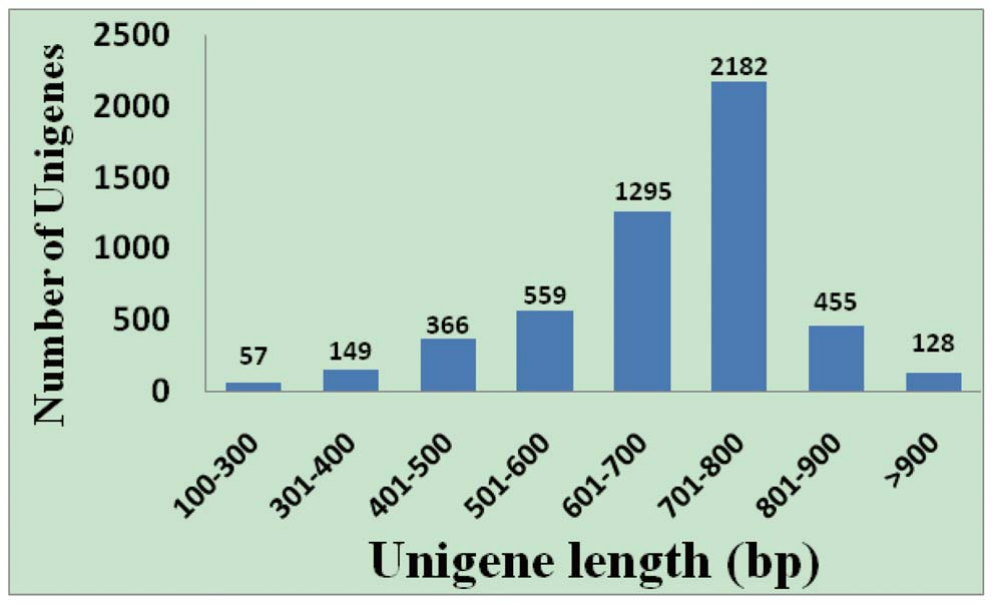
Sequence length distribution of upland cotton ESTs after assembly.

**Figure 2:**
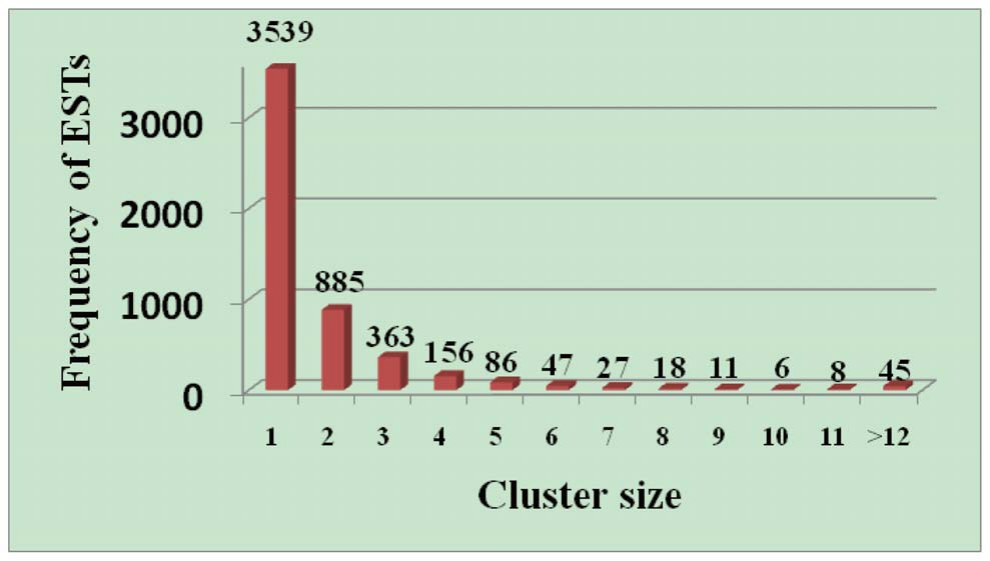
Frequency and distribution of ESTs among assembled contigs.

Approximately 11,623 clones were successfully single-pass sequenced from their 3′ ends. The insert sizes ranged from 900–3,000 bp, with an estimated average size of 1,200 bp. After removal of vector, poly(A) tails, contaminating microbial sequences, and those shorter than100 bp, 9,874 ESTs were considered high confidence (Q20) and were deposited in the GenBank dbEST database (JZ110066–JZ119939). Clustering and assembly of the ESTs were carried out under stringent conditions to obtain 5,191 putative unigenes, including 1,652 (31.8%) contigs that consisted of two or more ESTs and 3,539 (68.2%) singletons (Table 1). The EST redundancy of this library was 47.4%, and the unigenes had an average length of 682 bp. The distribution of high-quality EST sequences in the clusters is shown in Figure 1. Of the 1,652 contigs, 885 (53.6%) contained two ESTs, 363 (22.0%) contained three ESTs, 156 (9.4%) contained four ESTs, 86 (5.2%) contained five ESTs, 47 (2.8%) contained six ESTs, and relatively few (7.0%) contained more than six ESTs (Figure 2). The unigene mean size was only 1.9 sequences, and each contig averaged 3.8 sequences. These results also suggested that the redundancy rate of this normalized library was relatively low.

**Table 1:** Summary statistics of EST data generated from 11,623*Gossypium hirsutum* leaves.

Feature	Number
Total ESTs	11,623
High-quality ESTs	9,874
ContigsESTs in contigs	1,6526,335
Singletons	3,539
Unigenes	5,191
Average unigene sequence length (bp)	682.5

The most abundant ESTs are shown in Table 2 (each contig contained ≥10 EST copies). Some of these genes have important roles in leaf senescence. For example, lipid transfer protein precursors (Contig1797, Contig1794, Contig1792, and Contig1778; 97 ESTs) were involved in nutrient recycling for lipid transfer. Cysteine protease (Contig1795), polyubiquitin (Contig1784), putative serine carboxypeptidase precursor (Contig1749), and aspartyl protease (Contig1748) play roles in protein degradation. Peroxidase (Contig1766) is an antioxidant important for redox regulation, and metallothionein-like protein (Contig1756) is a low-molecular-weight Cys-rich protein that functions in heavy metal detoxification to remobilize valuable metal ions. Among these unigenes, 4,876 (93.9%) had open reading frames (ORFs) that were longer than 100 bp. The average ORF was 342 bp. The mean G/C content of unigenes was approximately 42%, which was approximately equivalent to that of *Arabidopsis* (43.2%) and much lower than that of rice (55.2%) [22], [23].

**Table 2:** The most abundant ESTs detected in the *Gossypium hirsutum* leaf library.

Unigene name	Number of unigenes	Putative function	Source organism	E-value
Contig1802	51	alpha-tubulin-1	*Pisum sativum*	1.00E-105
Contig1801	41	protodermal factor 1.1	*Gossypium barbadense*	3.00E-48
Contig1800	36	alpha-expansin 1	*Gossypium hirsutum*	1.00E-103
Contig1799	35	alpha-expansin 1	*Gossypium hirsutum*	5.00E-81
Contig1798	32	E6-2 protein kinase	*Gossypium barbadense*	3.00E-82
Contig1797	32	lipid transfer protein 4 precursor	*Gossypium hirsutum*	5.00E-48
Contig1796	31	membrane protein f16	*Gossypium hirsutum*	7.00E-98
Contig1795	30	cysteine protease Cp5	*Vitis vinifera*	2.00E-68
Contig1794	28	lipid transfer protein 3 precursor	*Gossypium hirsutum*	7.00E-54
Contig1793	22	chalcone isomerase	*Gossypium hirsutum*	7.00E-82
Contig1792	22	lipid transfer protein 3 precursor	*Gossypium hirsutum*	4.00E-52
Contig1791	20	anthocyanidin reductase	*Gossypium hirsutum*	1.00E-128
Contig1790	19	alpha-tubulin-1	*Pisum sativum*	1.00E-99
Contig1789	19	proline-rich protein	*Gossypium hirsutum*	5.00E-33
Contig1788	19	tubulin alpha chain	*Heterocapsa rotundata*	2.00E-83
Contig1787	19	Non-specific lipid-transfer protein 3	*Prunus dulcis*	5.00E-34
Contig1786	17	60S ribosomal protein L29	*Ricinus communis*	1.00E-27
Contig1785	17	glyceraldehyde-3-phosphate dehydrogenase C subunit	*Gossypium hirsutum*	1.00E-114
Contig1784	17	polyubiquitin	*Vitis vinifera*	1.00E-106
Contig1783	16	phosphoglycerate kinase	*Gossypium hirsutum*	1.00E-93
Contig1782	16	Hypothetical protein SELMODRAFT_178975	*Selaginella moellendorffii*	1.00E-103
Contig1781	16	3-ketoacyl-CoA synthase	*Gossypium hirsutum*	2.00E-86
Contig1780	16	S28 ribosomal protein	*Triticum aestivum*	3.00E-20
Contig1779	15	40S ribosomal protein S2	*Ricinus communis*	3.00E-94
Contig1778	15	lipid transfer protein precursor	*Gossypium hirsutum*	1.00E-52
Contig1777	15	hypothetical protein	*Vitis vinifera*	1.00E-105
Contig1776	15	arabinogalactan protein	*Gossypium hirsutum*	4.00E-77
Contig1775	14	tubulin beta-1	*Gossypium hirsutum*	9.00E-96
Contig1774	14	translation elongation factor 1A-2	*Gossypium hirsutum*	1.00E-103
Contig1773	14	no hit	–	–
Contig1772	14	high-glycine tyrosine keratin-like protein	*Gossypium hirsutum*	3.00E-48
Contig1771	14	Patellin-3	*Ricinus communis*	4.00E-77
Contig1770	14	histone H2B.2	*Camellia sinensis*	7.00E-43
Contig1769	14	protodermal factor 1.3	*Gossypium hirsutum*	9.00E-50
Contig1768	14	MELLADRAFT_87680	*Melampsora larici-populina*	1.00E-102
Contig1767	13	unnamed protein product	*Vitis vinifera*	2.00E-99
Contig1766	13	peroxidase	*Gossypium hirsutum*	3.00E-71
Contig1765	13	flavonoid 3′5′-hydroxylase	*Gossypium hirsutum*	1.00E-119
Contig1764	13	conserved hypothetical protein	*Ricinus communis*	2.00E-50
Contig1763	13	S-adenosyl-L-homocystein hydrolase	*Gossypium hirsutum*	1.00E-83
Contig1762	13	ARALYDRAFT_890328	*Arabidopsis lyrata subsp. lyrata*	2.00E-35
Contig1761	13	predicted protein	*Populus trichocarpa*	5.00E-77
Contig1760	12	S-adenosyl-L-homocystein hydrolase	*Gossypium hirsutum*	3.00E-74
Contig1759	11	intercellular adhesion molecule 2 precursor variant	*Homo sapiens*	4.00E-06
Contig1758	11	unknown	*Medicago truncatula*	6.00E-93
Contig1757	11	putative SAH7 protein	*Gossypium raimondii*	8.00E-63
Contig1756	11	metallothionein-like protein	*Gossypium hirsutum*	8.00E-27
Contig1755	11	cydophilin	*Gossypium hirsutum*	1.00E-86
Contig1754	11	gibberellin 20-oxidase 1	*Gossypium hirsutum*	2.00E-83
Contig1753	11	no hit	*-*	-
Contig1752	11	unnamed protein product	*Vitis vinifera*	1.00E-37
Contig1751	10	calreticulin	*Carica papaya*	8.00E-47
Contig1750	10	Superoxide dismutase [Mn]	*Prunus persica*	8.00E-76
Contig1749	10	putative serine carboxypeptidase precursor	*Gossypium hirsutum*	1.00E-122
Contig1748	10	aspartyl protease family protein	*Arabidopsis lyrata subsp. lyrata*	6.00E-66
Contig1747	10	heat shock protein 70	*Gossypium hirsutum*	1.00E-103
Contig1746	10	chalcone synthase 1	*Gossypium hirsutum*	3.00E-93

### Unigene Functional Annotation and Functional Categorization

To annotate the unigenes, all unigenes were used in a blastx search against the NCBI non-redundant (nr) protein database with a cut-off E-value of 10^−5^. The nr database is commonly used as the principal target database to search for homologous proteins. Using this approach, most unique sequences (84.4%) had matches in the nr database. However, 808 sequences had no hits. Of the best matches, 1,094 (25%) were to *Ricinus*, 1011 (23.1%) to *Vitis*, 975 (22.2%) to *Populus*, 158 (3.6%) to *Arabidopsis*, 142 (3.2%) to *Glycine*, 49 (1.1%) to *Medicago*, and 27 (0.6%) to *Oryza*, whereas only 490 (11.2%) of the best matches were to cotton (Table 3). Comparison of our unigene data set with the NCBI nucleotide database using blastn demonstrated that 4,138 unigenes (79.7%) had significant matches. All unigenes were also blastx searched against the Swiss-Prot database, in which 2,973 (57.3%) unigenes matched. The best hits were mainly to *Arabidopsis* (1,514 hits, 50.9%) and rice (151 hits, 5.1%).

**Table 3:** Comparison of the *Gossypium hirsutum* leaf EST library with those of other species.

Species	Number of unigenes	Percentage
*Ricinus*	1094	25.0%
*Vitis*	1011	23.1%
*Populus*	975	22.2%
*Gossypium*	490	11.2%
*Arabidopsis*	158	3.6%
*Glycine*	142	3.2%
*Medicago*	49	1.1%
*Homo*	37	0.8%
*Jatropha*	34	0.8%
*Oryza*	27	0.6%
*Citrus*	19	0.4%
*Cucumis*	16	0.4%
*Malus*	14	0.3%
*Nicotiana*	14	0.3%
*Picea*	14	0.3%
*Prunus*	14	0.3%
*Solanum*	14	0.3%
*Pisum*	12	0.3%
*Sorghum*	12	0.3%
*Zea*	12	0.3%
Others	104	5%

**Figure 3:**
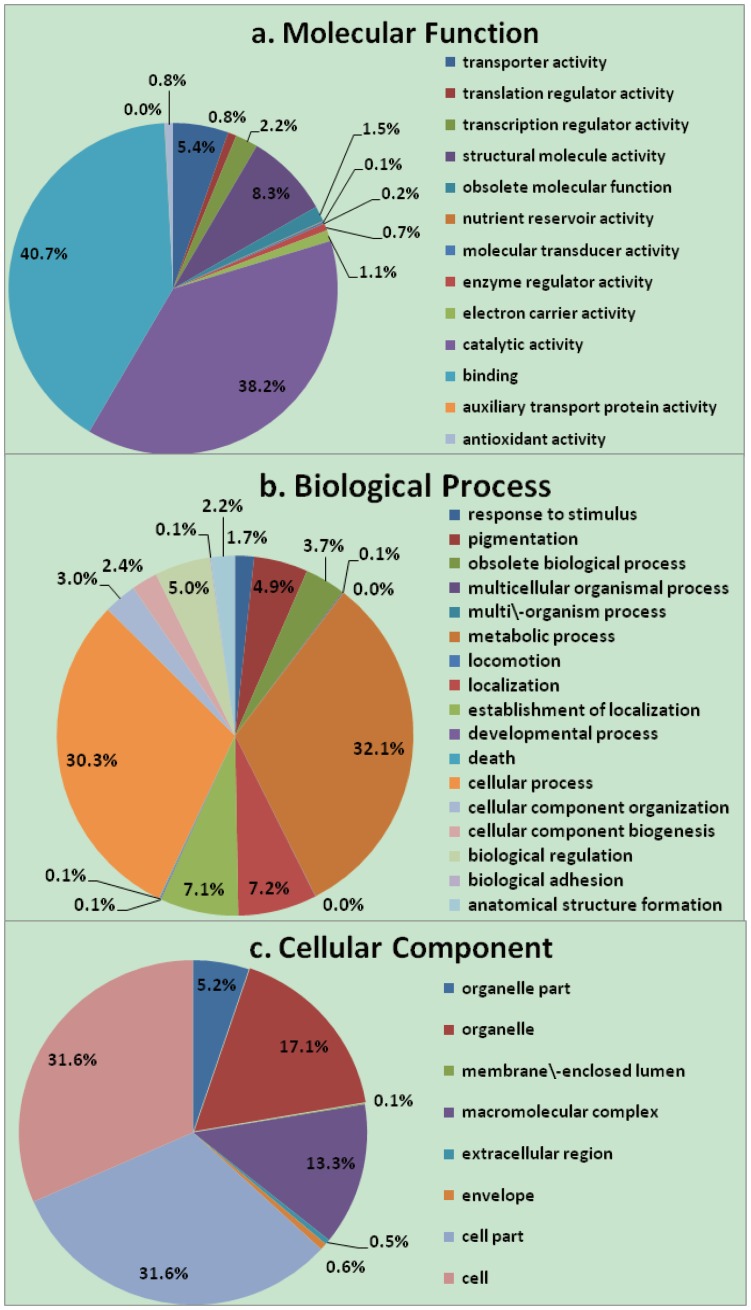
Functional classifications of upland cotton 2,416 unigenes that were assigned with GO terms. Three GO categories are presented: (a) molecular function, (b) biological process, and (c) cellular component.

GO analysis has been widely used to classify gene functions [24]. In total, 2,416 (46.6%) unigenes fell into one or more of these categories: molecular function (2,147; 41.4%), cellular component (953; 18.4%), and biological process (1,757; 33.8%) (Figure 3). Within the molecular function category, most unigenes were assigned to molecular transducer activity (40.7%), catalytic activity (38.2%), and structural molecular activity (8.3%). The largest proportion of functionally-assigned contigs in the biological process category was categorized as metabolic process (32.1%), cellular process (30.3%), localization (7.2%), establishment of localization (7.1%), and biological regulation (5.0%). In the cellular component category, the most highly-represented groups were cell part (31.6%), cell (31.6%), and organelle (17.1%). Protein families, domains, and functional sites for the *G. hirsutum* unigenes were obtained through InterProScan. The most common InterPro families are presented in Table 4. A total of 3,199 unigenes fell into 1,150 InterPro families. The most frequent family was protein kinase, core (IPR000719), with 89 unigenes, followed by zinc finger, RING-type (IPR001841, 41 unigenes), WD40 repeat (IPR001680, 36 unigenes), beta tubulin (IPR000217, 30 unigenes), cytochrome P450 (IPR001128, 29 unigenes), and RNA recognition motif, RNP-1 (IPR000504, 29 unigenes).

**Table 4:** Fifty most frequent InterPro families found in the *Gossypium hirsutum* leaf EST library.

No.	Interpro no.	Description	Number of unigenes
1	IPR000719	Protein kinase, core	89
2	IPR001841	Zinc finger, RING-type	41
3	IPR001680	WD40 repeat	36
4	IPR000217	Beta tubulin	30
5	IPR001128	Cytochrome P450	29
6	IPR000504	RNA recognition motif, RNP-1	29
7	IPR000886	Endoplasmic reticulum targeting sequence	25
8	IPR000608	Ubiquitin-conjugating enzyme, E2	25
9	IPR002048	Calcium-binding EF-hand	22
10	IPR001806	Ras GTPase	22
11	IPR002198	Short-chain dehydrogenase/reductase SDR	21
12	IPR000637	HMG-I and HMG-Y, DNA-binding	21
13	IPR000626	Ubiquitin	21
14	IPR000528	Plant lipid transfer protein/Par allergen	19
15	IPR001993	Mitochondrial substrate carrier	18
16	IPR015706	RNA-directed DNA polymerase (reverse transcriptase), related	17
17	IPR003959	AAA ATPase, core	17
18	IPR001087	Lipolytic enzyme, G-D-S-L	17
19	IPR004160	Translation elongation factor EFTu/EF1A, C-terminal	16
20	IPR002085	Alcohol dehydrogenase superfamily, zinc-containing	16
21	IPR000425	Major intrinsic protein	16
22	IPR004000	Actin/actin-like	15
23	IPR001464	Annexin	15
24	IPR001461	Peptidase A1	15
25	IPR000727	Target SNARE coiled-coil region	15
26	IPR005123	2OG-Fe(II) oxygenase	14
27	IPR001813	Ribosomal protein 60S	14
28	IPR001353	20S proteasome, A and B subunits	14
29	IPR003612	Plant lipid transfer protein/seed storage/trypsin-alpha amylase inhibitor	13
30	IPR001611	Leucine-rich repeat	13
31	IPR000717	Proteasome component region PCI	13
32	IPR000169	Peptidase, cysteine peptidase active site	13
33	IPR004159	Protein of unknown function DUF248, methyltransferase putative	12
34	IPR004853	Protein of unknown function DUF250	11
35	IPR003388	Reticulon	11
36	IPR002423	Chaperonin Cpn60/TCP-1	11
37	IPR002213	UDP-glucuronosyl/UDP-glucosyltransferase	11
38	IPR001023	Heat shock protein Hsp70	11
39	IPR001005	SANT, DNA-binding	11
40	IPR007493	Protein of unknown function DUF538	10
41	IPR004240	Nonaspanin (TM9SF)	10
42	IPR003311	AUX/IAA protein	10
43	IPR002052	N-6 Adenine-specific DNA methylase	10
44	IPR000308	14-3-3 protein	10
45	IPR015590	Aldehyde dehydrogenase	9
46	IPR004045	Glutathione S-transferase, N-terminal	9
47	IPR003439	ABC transporter related	9
48	IPR002109	Glutaredoxin	9
49	IPR002016	Haem peroxidase	9
50	IPR001509	NAD-dependent epimerase/dehydratase	9

### Comparison with Previous Cotton ESTs

To evaluate potential novel sequences that did not match to sequences from other cotton species in the databases, the unigenes were used as queries in a blastn search against the Dana-Farber Cancer Institute (DFCI) Cotton Gene Index database. Approximately 24.3% of the ESTs and 19.1% unique sequences generated in this study were not highly homologous to known cotton ESTs or unique sequences. Thus, our library provides a valuable new transcript resource, with 2,400 new ESTs and 991 new unique sequences for cotton.

### Identification and Analysis of Leaf Senescence-related Protein Families

To identify leaf SAGs, all the unigenes were assessed by blastx against the amino acid sequences of *A. thaliana* genes from the leaf senescence database (LSD). Of the 5,191 unigenes, 1,339 (25.8%) had homologs matched with 455 (44.6%) SAGs of *A. thaliana* in the LSD, and could be classified into 29 leaf senescence-related categories (Table 5). The most abundant leaf senescence-related category was protein degradation/modification, with a total of 199 unigenes. Other highly-abundant leaf SAGs included Nutrient recycling, Lipid/Carbohydrate metabolism, Signal transduction, Transcriptional regulation, Redox regulation, Stress and detoxification, and Hormone response pathway. These functions are all closely involved with leaf senescence.

**Table 5:** Functional categories of *Gossypium hirsutum* leaf senescence-related genes^a^.

Function	Totalunigenes	TotalESTs	Redundancy
Protein degradation/modification	199	373	1.9
Nutrient recycling	168	454	2.7
Lipid/Carbohydrate metabolism	158	338	2.1
Signal transduction	147	248	1.7
Transcriptional regulation	133	210	1.6
Redox regulation	94	267	2.8
Stress and detoxification	51	124	2.4
Hormone response pathway	49	70	1.4
Defense	22	28	1.3
Cell structure	22	30	1.4
Nucleic acid degradation	19	36	1.9
Detoxification	9	23	2.6
Metal binding	7	12	1.7
ATPases	6	12	2.0
Metabolism	4	12	3.0
Secondary metabolites	3	4	1.3
Chlorophyll degradation	2	8	4.0
Zinc finger protein	2	2	1.0
snRNP	2	4	2.0
Light signal	2	4	2.0
Dioxygenase	2	4	2.0
Others	236	386	1.6
Total	1337	2649	2.0

a Frequency of unigenes found in the present study withsignificant similarities to *Arabidopsis thaliana* genes in the leaf senescence database.

**Figure 4:**
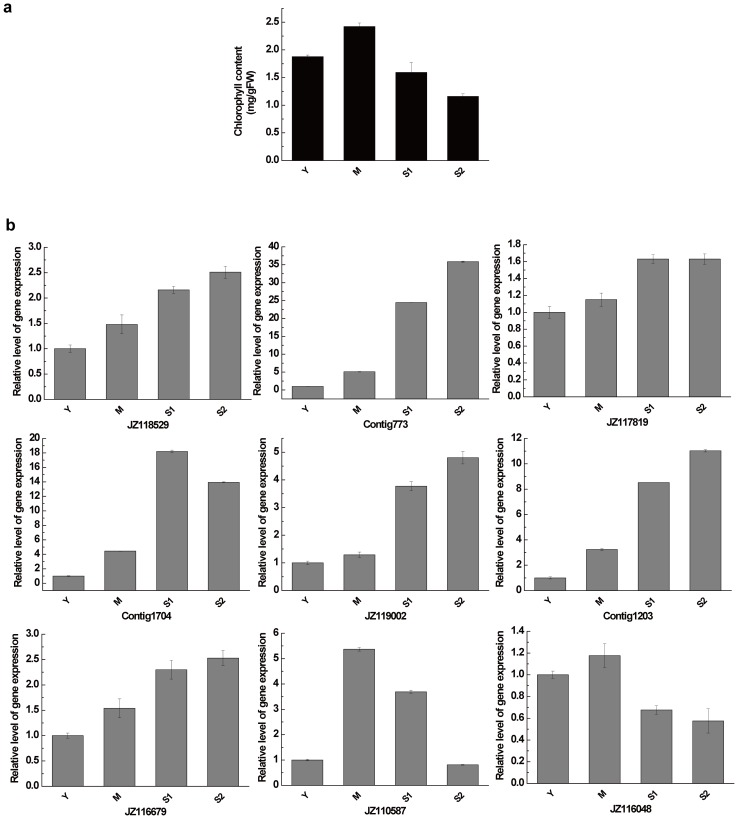
Expression patterns of nine putative leaf senescence related genes from upland cotton. (a) Chlorophyll contents per fresh weight of leaves at each of four developmental stages. (b) Changes in transcript levels of the nine putative leaf senescence-related genes at each leaf developmental stage.

To study the expression of genes associated with leaf senescence, first, representative leaves were classified as young leaves (Y), mature leaves (M), early-senescent leaves (S1) and late-senescent leaves (S2) by their chlorophyll contents, as shown in Figure 4a. The chlorophyll content in S1 and S2 was 65% and 45% of that in M, respectively. Then, nine putative leaf senescence-related ESTs were randomly selected for qRT-PCR using RNA isolated from leaves of those four stages. Most of these ESTs were up-regulated during senescence, especially Contig773, whose expression level increased significantly in the late senescent leaves (Figure 4b). Only two ESTs, JZ110587 and JZ116048, were down-regulated. Characterization of these potential regulatory genes provided clues to the regulatory mechanism of leaf senescence.

### Identification and Analysis of Putative Transcription Factors

*Arabidopsis thaliana* TFs, including 2023 TFs and 58 families, in the comprehensive PlantTFDB 2.0 database [25], were used to identify putative TFs in the cotton EST collection. Blastx searches revealed 200 (4.8% of unigenes) with matches to *Arabidopsis* (E-value ≤10^−10^) (Table 6). These TFs fell into 41 families. The most abundant family was the MYB group (22 unigenes, 11.0%), followed by bHLH (17, 8.5%), bZIP (16, 8.0%), C3H (13, 6.5%), NAC (11, 5.5%), ERF (10, 5.0%), ARF (9, 4.5%), C2H2 (9, 4.5%), and WRKY (9, 4.5%).

**Table 6:** The most abundant putative transcriptional factors (TFs).

TF family	TF description	Total of unigenes	Percent (%)^a^
MYB	Myb-like DNA-binding domain	22	11.0%
bHLH	basic/helix-loop-helix domain	17	8.5%
bZIP	Basic leucine zipper (bZIP) motif	16	8.0%
C3H	Zinc finger, C-x8-C-x5-C-x3-H type	13	6.5%
NAC	No apical meristem (NAM) protein	11	5.5%
ERF	single AP2/ERF domain	10	5.0%
ARF	Auxin response factor	9	4.5%
C2H2	Zinc finger, C2H2 type	9	4.5%
WRKY	WRKY DNA-binding domain	9	4.5%
MIKC	MIKC-type MADS-box gene include three more domains intervening (I) domain,keratin-like coiled-coil (K) domain, and Cterminal (C) domain	6	3.0%
TCP	TCP domain	6	3.0%
CO-like	CONSTANS like	5	2.5%
HB-other	Homeobox domain	5	2.5%
HD-ZIP	HD domain with a leucine zipper motif	5	2.5%
G2-like	Golden 2-like (GLK)	4	2.0%
GATA	one or two highly conserved zinc finger DNA-binding domains	4	2.0%
GRAS	three initially identified members, GAI, RGA and SCR	4	2.0%
Trihelix	Trihelix DNA-binding domain	4	2.0%
ARR-B	*Arabidopsis* response regulators(ARRs) with a Myb-like DNA binding domain(ARRM)	3	1.5%
Dof	DNA binding with one zinc finger	3	1.5%
SBP	SBP-domain	3	1.5%
ZF-HD	zinc finger homeodomain	3	1.5%
Other	–	29	14.5%

a Percent = (total number of unigenes)/(total number of putative TFs). There were 200 putative TFs.

**Figure 5:**
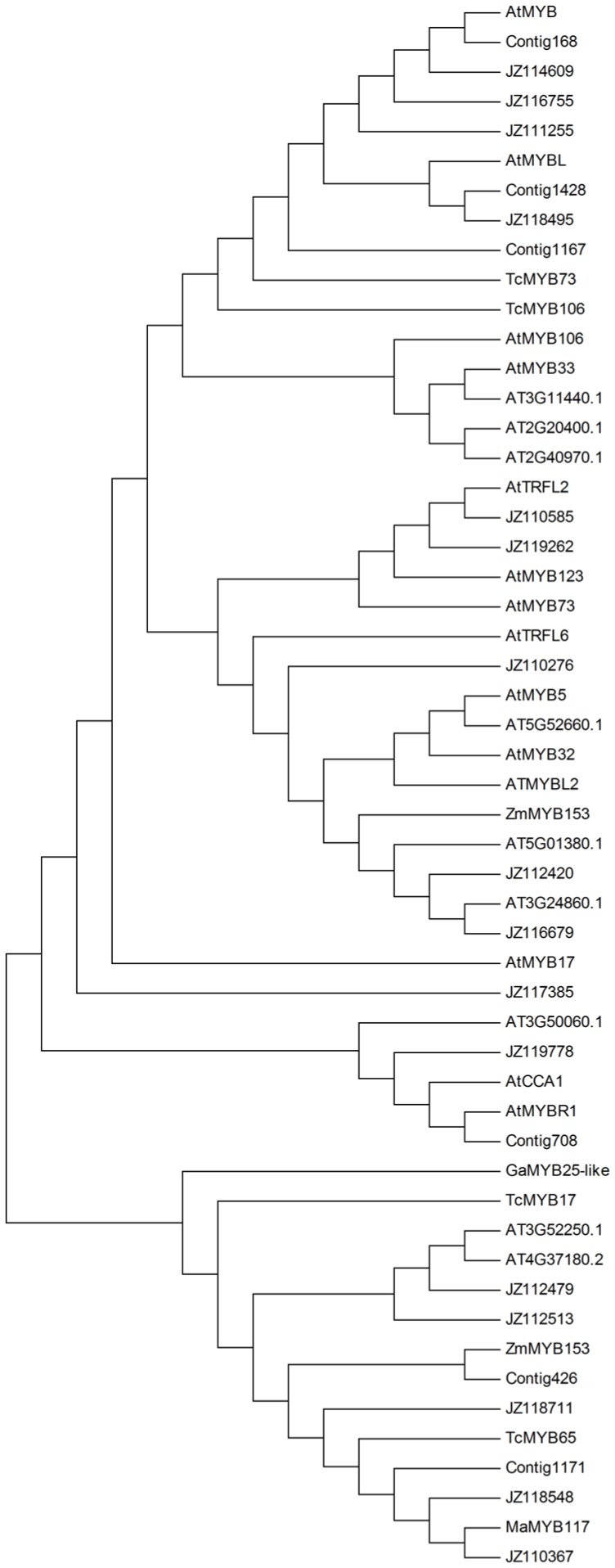
Phylogeny analysis of putative MYB transcription factors. Twenty-two putative cotton MYB transcription factors and thirty-one putative MYB transcription factors from other plant species were aligned and analyzed by neighbor-joining in MEGA4.

**Figure 6:**
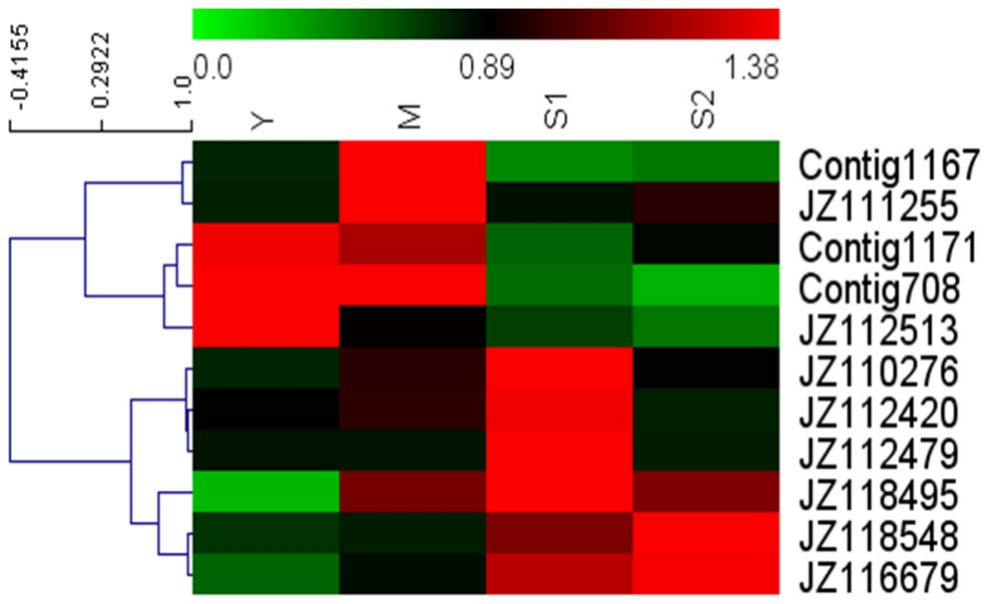
Expression patterns of 11 qRT-PCR was used to evaluate the relative levels of these ESTs at each leaf development stage. The patterns were clustered and viewed using software MeV4.7.4.

To identify the potential roles of these TFs during leaf senescence, the most abundant MYB family in this normalized library was selected and its expression pattern was analyzed. As shown in Figure 5, several putative cotton MYB orthologs matched *AtMYBL* (AT1G49010), *ZmMYB153* (GRMZM2G050550), *AtMYB* (AT4G01280), AT3G24860.1, *AtMYBR1* (AT5G67300), AT3G52250.1, and AT4G37180.2, which play roles in leaf senescence [26]–[28]. Using qRT-PCR, we confirmed the transcript abundance of selected ESTs encoding putative MYB TFs in leaves at different developmental stages (Figure 6). JZ118495, JZ116679 and JZ112479 were putative cotton orthologs of *AtMYBL* (AT1G49010), AT3G24860.1 and AT4G37180.2, respectively, from *A. thaliana* (Figure 5). The expression of JZ118495 increased in M stage and reached a maximum in S1 stage, while that of JZ116679 increased gradually during leaf senescence and peaked in S2 stage, and JZ112479 was expressed at high levels in the S1 stage but at reduced levels in the S2 stage (Figure 6). Six of 11 ESTs were highly expressed in senescent leaves; most increased in the expression level in the S1 stage, including JZ110276, JZ112420, JZ112479, and JZ118495. Other transcripts, such as Contig1167, JZ111255, Contig 1171, Contig708 and JZ112513 were down-regulated during leaf senescence. The results indicated that these MYB TFs may be involved in controlling leaf senescence in cotton.

### Cloning of Upland Cotton YLS Homologous Genes: Sequence, Phylogenetic, and Expression Analyses

**Figure 7:**
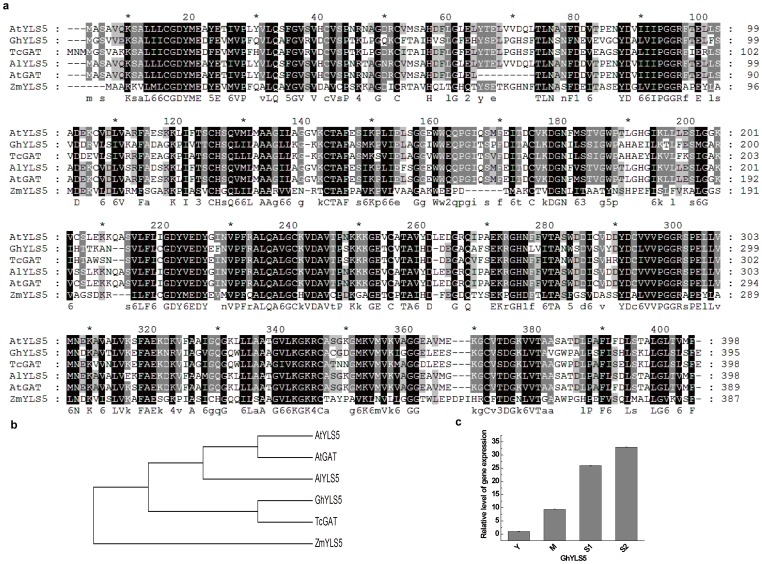
Analysis of GhYLS5 relationships. (a) Multiple sequence alignment of GhYLS5 and other homologous proteins in plants: *Theobroma cacao* GAT (EOX94596), *Arabidopsis thaliana* GAT (NP_850303), *A. thaliana a* YLS5 (AB047808), *Arabidopsis lyrata* YLS5 (XP_002881620), and *Zea mays* YLS5 (NP_001146927). (b) Phylogenetic tree of these plant proteins constructed with MEGA 4 (c) Changes in transcript levels of GhYLS5 genes at each leaf development stage.

**Figure 8:**
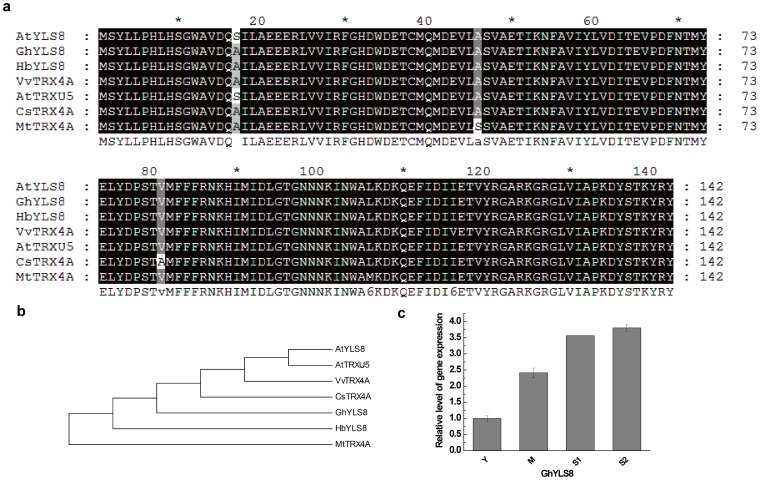
Analysis of GhYLS8 relationships. (a) Multiple sequence alignment of GhYLS8 and other homologous proteins in plants: *Arabidopsis thaliana* YLS8 (AB047811), *Hevea brasiliensis* YLS8 (XP_004148041), *Cucumis sativus* TRX4A (XP_004163626), *Medicago truncatula* TRX4A (XP_003590204), *A. thaliana* TRXU5(AED91278) and *Vitis vinifera* TRX4A (XP_002310072). (b)Phylogenetic tree of these plant proteins constructed with MEGA 4 (c) Changes in the transcript levels of GhYLS8 genes at each leaf development stage.

**Figure 9:**
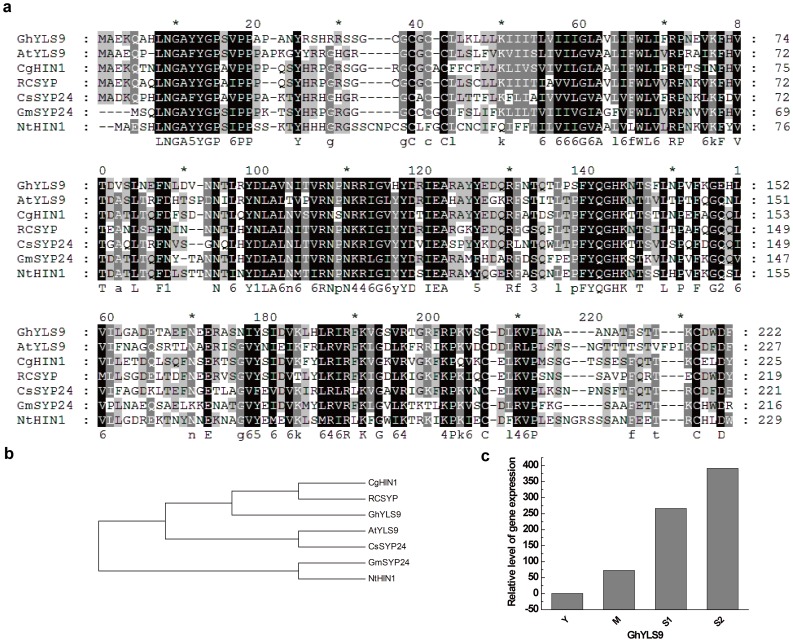
Analysis of GhYLS9 relationships. (a) Multiple sequence alignment of GhYLS9 and other homologous proteins in plants: *Arabidopsis thaliana* YLS9 (AB047812), *Casuarina glauca* HIN1 (ABZ80409), *Nicotiana tabacum* HIN1 (BAD22533), *Ricinus communis* SYP(XP_002532540), *Cucumis sativus* SYP24 (XP_004136508) and *Glycine max* SYP24 (XP_003554459). (b) Phylogenetic tree of these plant proteins constructed with MEGA 4 (c) Changes in transcript levels of GhYLS9 genes at each leaf development stage.

To confirm that our full-length library was an efficient method for rapid functional gene discovery in upland cotton, three *A. thaliana* homologs of yellow-leaf-specific genes (*YLS*) were cloned and analyzed. The *Arabidopsis* YLS proteins were used as queries to search our EST database with tBLASTn. Three unique full-length sequences were found in upland cotton and named *GhYLS5* (JX163920), *GhYLS8* (JX163921), and *GhYLS9* (JX163922). In *A. thaliana*, the *YLS5* gene encoded a proteaseI (pfpI)-like protein of 398 amino acid residues that was expressed weakly in young leaves and strongly in senescent leaves. This gene can be induced by artificial senescence processes such as darkness, ethylene, and ABA treatment [15]. The *GhYLS5* gene had an ORF of 1,188 bp and encoded a protein of 395 amino acid residues. Multiple sequence alignment showed that GhYLS5 proteins were homologous to the glutamine amidotransferase (GAT) of *A. thaliana* and *Theobroma cacao* and with YLS5 of *A. thaliana*, *Arabidopsis lyrata* and *Zea mays* with identities of 51–84% (Figure 7a, b). *Arabidopsis YLS8* contained an ORF encoding a Dim1 homolog of 142 amino acid residues that had high expression in senescent and virus-infected leaves [15], [29]. The *GhYLS8* gene had an ORF of 429 bp, encoding a protein of 142 amino acid residues. The protein of GhYLS8 was highly conserved, with very high sequence homology to YLS8 from *A. thaliana*, *Hevea brasiliensis*, *Matthiola longipetala*, *Iberis amara*, and *Lepidium sativum* and to thioredoxin-like protein 4A (TRX4A) from *A. thaliana*, *Cucumis sativus*, *Vitis vinifera* and *Medicago truncatula* (Figure 8a, b). The *YLS9* gene (also called *NHL10*) of *Arabidopsis* contained an ORF encoding a polypeptide of 227 amino acid residues, whose sequence was similar to tobacco hairpin-induced gene (*HIN1*) and *Arabidopsis* non-race specific disease resistance gene (*NDR1*). Expression of this gene is induced by *Cucumber* mosaic virus, spermine, and senescence [15], [29], [30].*GhYLS9* gene had an ORF of 669 bp, encoding a protein of 222 amino acid residues. GhYLS9 proteins were homologous to syntaxin (SYP) from *Ricinus communis*, *Cucumis sativus* and *Glycine max*, HIN1 from *Casuarina glauca* and *Nicotiana tabacum*, and YLS9 from *A. thaliana*, with identities of 51–62% (Figure 9a, b). The expression of three *GhYLS* transcripts were also analyzed using qRT-PCR at different leaf developmental stages (Figures 7c, 8c, 9c). The three genes were all up-regulated in senescent leaves. In particular, expression of *GhYLS9* was nearly 400-fold higher than in young leaves. These results suggested that leaf senescence related-genes could be identified from our library using -homologous sequence searches.

## Discussion

*Gossypium hirsutum* is one of the most economically-important species in its genus. Unfortunately, to date, its genome has not been completely sequenced. Recent efforts have demonstrated that EST sequencing is an efficient and relatively low-cost approach for large-scale gene discovery, annotation, and comparative genomics research [31]. In *G. hirsutum*, although many ESTs are available, the total number is less than that of some field crops and model plants, and most ESTs in GenBank are from fibers or fiber-bearing ovules [11], [32]–[34] and provide little or no information regarding leaf development. Therefore, *G. hirsutum* leaf ESTs must be sequenced to examine the functional genomics of cotton leaf development. In this study, we produced 9,874 high-quality ESTs that assembled into 5,191 unigenes from a normalized leaf cDNA library. The leaf samples spanned all development stages, including unexpanded young leaves, fully-expanded mature leaves, and senescent leaves, at the plant blooming stage. This is the first such database and largest number of unique sequences from *G. hirsutum* leaf tissues to include all developmental periods. This EST resource provides a foundation for molecular control of *G. hirsutum* leaf growth and development and for future whole-genome sequencing and analysis of the functional genome and gene expression patterns.

Normalized cDNA libraries overcome problems caused by differential expression of genes and are an efficient and cost-effective tool for obtaining large-scale unique EST sequences and for gene identification [35]. Our cDNA library was normalized by saturation hybridization with genomic DNA, assuming relatively uniform copy numbers of most of genes in the genome. EST assembly revealed a novelty rate of 52.6%, a redundancy rate of 47.4%, and 68.2% of unigenes that contained only one EST. Thus, there remains considerable potential to discover additional novel sequences by sequencing randomly-selected cDNAs from this library. Alpha-tubulin 10 (TUA10) and ubiquitin (UBI1), the most redundant transcripts in cotton leaves, were represented by only 19 and 17 clones in our ESTs, respectively. Furthermore, the copies of two highly abundant genes actin and 18S, decreased 145 and 200 fold after cDNA library was normalized, respectively. These results reflect the quality of the normalized library and also showed that this approach was an efficient tool for gene identification because it reduced variation among abundant clones and increased the probability of sequencing rare transcripts.

The majority of annotated sequences with BLAST hits were transcripts from the rosid clade, to which cotton (Malvales; eurosids II clade) also belongs. *Ricinus* (25% of the best matches) and *Populus* (22.2%) belong to the eurosids I clade, while *Vitis* (23.1%) is a basal rosid [36]. Although *A. thaliana* and *O. sativa* are well-studied model systems with completely-sequenced genomes, these organisms were best matches to only 3.6% and 0.6% of our unique sequences, respectively. Yu [37] investigated the conservation of colinearity between cotton BAC sequences and other model plant genomes; on a phylogeny of single-copy orthologous genes from cotton, *Arabidopsis*, poplar, grape, rice, and maize, poplar was the closest relative to cotton. *Arabidopsis thaliana*, *P. trichocarpa*, and *G. hirsutum* are dicots, while *O. sativa* is a monocot, which may account for the differences in similarity among their sequences. Only 11.2% of the hits were to cotton sequences already available in GenBank, highlighting the lack of sequence information for this genus and the value of our EST sequences. Clearly, genome sequencing of *G. hirsutum* represents a vital and urgent need. Furthermore, we discovered 2,400 new cotton ESTs and 991 unique cotton sequences when comparing our data to the DFCI Cotton Gene Index database. Our data will contribute to the enrichment of cotton genetic and physical maps.

In previous studies, much attention was focused on leaf senescence, especially in *Arabidopsis* and rice [18], [38]–[42]. Leaf senescence constitutes the last stage of leaf development and strongly affects cotton yield. Currently, however, the dynamic regulatory mechanisms of leaf senescence in cotton remain unclear. A large number of SAGs have been identified in various plants through microarray analyses [21], [43]. Some of them have been found to be TFs belonging to several different families, especially NAC, WRKY, C2H2-type zinc finger, AP2/EREBP, and MYB protein families [44], [45]. Characterization of these potential regulatory genes led to discovery of a few important senescence regulatory genes and provided some insight into the regulatory mechanism of leaf senescence. Using data from the PlantTFDB 2.0 database, we found 200 unigenes from our library that had high similarity with 163 TFs from 41 families. The most well-represented TF family in our library was the MYB group, followed by the bHLH, bZIP, C3H, NAC, ERF, ARF, C2H2, and WRKY families. Analysis of the expression patterns of several putative MYB transcript families showed that the expression level of some transcripts changed significantly during leaf senescence. This result indicated that some MYB TFs may play roles during leaf senescence. These results also were in accordance with those of previous studies. In addition to these TF families, several others known to be involved in plant development were also present in our data.

Leaf senescence is an integrated response of leaf cells to age and other internal and environmental signals. It is an exceptionally complex and dynamic genetic process [46]. *Arabidopsis thaliana* is a favorite model for the molecular genetic study of leaf senescence [47]–[49]. The LSD is also a platform to study leaf senescence [50]. Of the unigenes in our library, 1,339 could be classified into 29 SAG categories by a BLAST search against *A. thaliana* senescence-related proteins (1,021), such as nutrient recycling, Lipid/Carbohydrate metabolism, and hormone response pathway. During leaf senescence, nutrients in the leaf are reallocated to younger leaves, growing seeds, or other growing organs in a process of nutrient salvage, e.g., hydrolysis of macromolecules and subsequent remobilization, which requires complex array of metabolic pathways [51]. Many the genes involved in lipid metabolism function in leaf senescence. Lipid-degrading enzymes, such as lytic acyl hydrolase, phosphatidic acid phosphatase, phospholipase D, and lipoxygenase appear to be involved in hydrolysis and metabolism of the membrane lipid in senescing leaves [51], [52]. Changed expression of the *Arabidopsis* acyl hydrolase gene in transgenic plants led to altered leaf senescence phenotypes [53]. The hormonal pathways appear to affect all stages of leaf senescence. In this work, numerous genes belonging to hormone response pathways were also identified. These results indicated that many previously-known leaf SAGs and pathways were included in this library. Three *GhYLS* genes were successfully cloned and analyzed. Their expression profiles revealed that their transcripts accumulated in leaves during senescence. Thus, these genes could potentially serve as molecular markers for distinguishing the complex regulatory networks of leaf senescence processes. This library provides a robust sequence resource and will be a useful tool for cloning the full-length sequences of functional genes for further leaf senescence-related analysis in *G. hirsutum*.

## Materials and Methods

### Plant Material

Upland cotton CCRI 36 (a short-season cultivar) was grown on the experimental farm of the Cotton Research Institute of Chinese Academy of Agricultural Sciences, Anyang, Henan Province. At the blooming stage, unexpanded leaves of the same size near the tops of stems were selected and marked. The day when leaves were fully expanded was considered the first day. Leaves were collected every 5 d for 70 d. Samples from each time point were pooled from at least 10 plants, frozen immediately in liquid nitrogen, and stored at –80°C.

### RNA Isolation and cDNA Library Construction

Total RNA was isolated by an improved CTAB method [54], and equal amounts of total RNA sampled at different time points were mixed to construct a full-length normalized cDNA library. Purification of mRNA from total RNA was carried out using the FastTrack® 2.0 Kit (Invitrogen, Carlsbad, CA, USA) following the manufacturer’s protocol. cDNAs were synthesized using the Superscript Full-length Library Construction Kit II (Invitrogen) according to the manufacturers’ protocols, cloned into a Gateway pDONR222 vector (Invitrogen) by the BP cloning process, and transformed into *Escherichia coli* strain DH10B competent cells (Invitrogen) through electroporation using an *E. coli* Pulser (BTX Harvard Apparatus, Holliston, MA, USA). After the full-length library was constructed, plasmid DNA was extracted with the PureLink™ HQ Mini Plasmid DNA Purification Kit (Invitrogen). Normalization was performed by saturation hybridization between genomic DNA and mixed plasmid DNA from the cDNA library [55]. Then, clones were randomly selected and fully sequenced to test fullness ratios of the cDNA inserts of the library. Putative full-length cDNA sequences were identified by comparison with all available ORF-complete mRNA sequences from the NCBI nr protein database [56]. Finally, qRT-PCR was used to estimate the relative concentration of a highly abundant clone in both the non-normalized and the normalized cDNA populations.

### EST Sequencing, Editing, and Assembly

Clones were randomly picked and transferred into 384-well plates. Selected clones were sequenced from the 3′ end on an ABI 3730 automatic DNA sequencer (Applied Biosystems, Foster City, CA, USA) using the M13 universal primer (M13R:CAGGAAACAGCTATGACC) and the BigDye Terminator Cycle Sequencing Kit (ABI) at the Invitrogen Sequencing Center. All sequences were clustered using the Phred/Phrap/Consed software package [57], [58]. The 3′ DNA EST sequence chromatogram files were base-called and quality trimmed (low-quality bases with<Q20 and <99% accuracy were removed) using Phred. Crossmatch (http://www.phrap.org/) and Repeat-Masker (http://www.repeatmasker.org/) were used to remove vector sequences and to identify and mask repeat sequences. Contaminating microbial sequences were eliminated using VecScreen (http://www.ncbi.nlm.nih.gov/VecScreen/VecSc-reen.html), and poly(A) tails were deleted. Sequences that passed the quality control screening for high-confidence base calls (Q20) and with lengths longer than 100 bp were defined as high quality EST and deposited into the dbESTs division of GenBank. The processed EST sequence files were combined and assembled into contigs and singlets (unisequences) using Phrap with a high stringency level (95% sequence identity with 20 bp overlap).

To validate potential novel ESTs and unique sequences that did not match any sequences in related cotton species in the existing databases, all the high-quality ESTs and assembled unigenes were compared against ESTs and unigenes already available in the DFCI Cotton Gene Index (http://compbio.dfci.harvard.edu/cgi-bin/tgi/gimain.pl?gudb=cotton) database, which contains 351,954 cotton ESTs and 2,315 ETs fully assembled into 117,992 unique sequences. With such stringent criteria, an EST was considered as new if it had at least 10% of its sequence with less than 95% of identity to any other EST or unigene in the public EST database.

### Prediction of ORFs, Unigene Functional Annotation, and Functional Categorization

All unique sequences were searched for putative ORFs with the Getorf program of EMBOSS-4.1.0 [59], and the longest sequences were used for functional analysis. Unigenes were compared with a variety of databases, including the NCBI non-redundant nucleotide and non-redundant protein databases, and Swiss-Prot, using either blastn (E-value≤10^−5^) or blastx (E-value≤10^−5^) [60]. To identify putative leaf SAGs and TFs, blastx (E-value≤10^−5^) searches against amino acid sequences of *A. thaliana* genes from a leaf senescence database (LSD) [55], [61] and a comprehensive plant TF database (PlantTFDB) [25] were used. Batch searches of the unigenes were performed using the local BLAST tools available at ftp://ftp.ncbi.nlm.nih.gov/blast/executables/blast/LATEST/. To assign GO terms, functional annotation was performed using Blast2GO software based on sequence similarity [62]–[64]. Furthermore, to improve annotations, results from an InterProScan search [65] (http://www.ebi.ac.uk/interpro/index.html) were merged with GO annotations and searched in the BlastProDom, FPrint-Scan, HMMPIR, HMMPfam, HMMSmart, HMMTigr, ProfileScan, ScanRegExp, and SuperFamily databases.

### Leaf Senescence Related Homolog Identification and Expression Pattern Analysis

To examine gene expressions during leaf development, the leaves used for qRT-PCR were harvested from approximately 10 individual plants for each stage. Total chlorophyll of the samples was measured as described by Lichtenthaler (1987) [66].Homologs of leaf senescence-related protein sequences were identified and randomly selected according to the LSD function annotation. Total RNA was extracted by an improved CTAB method as described above. cDNA was reverse transcribed from RNA by PrimeScript® RT Reagent Kit with gDNA Eraser (Takara, Otsu, Japan) with an Oligo dT Primer and random six-mers as the RT primer according to the manufacturer’s protocol. The specific primer pairs for nine selected genes and the internal control gene actin are listed in Table S1. qRT-PCR was performed with the SYBR Green PCR Master Mix (Takara) as recommended by the manufacturer in an ABI 7500 Real-time PCR System (Applied Biosystems) with three replicates. To analyze changes in gene expression, values from triplicate real-time PCRs were normalized to the expression level of actin and to the Y sample by the 2^–ΔΔCt^ method [67]. *Arabidopsis YLS* genes were used as queries to tBLASTn search against the cDNA library. The identified clones were sequenced in both directions with the internal primers. The amino-acid multiple-sequence alignment was analyzed using GeneDoc. Phylogenetic analysis was performed using the neighbor-joining method in MEGA 4 [68]. Expression patterns were detected by qRT-PCR as described above.

## Supporting Information

Table S1 **Primers used in gene-specific qRT-PCR of leaf senescence related genes.**
(DOC)
