# Supplementary material for: Generation and Analysis of a Large-Scale Expressed Sequence Tag Database from a Full-Length Enriched cDNA Library of Developing Leaves of Gossypium hirsutum L
Source: PLoS One. 2013 Oct 11;8(10):e76443. doi: 10.1371/journal.pone.0076443 (PMC3795732; doi:10.1371/journal.pone.0076443)
Supplement: Table S1 — Primers used in gene-specific qRT-PCR of leaf senescence related genes. (DOC) [file pone.0076443.s001.doc]

**Table S1 Primers used in gene-specific qRT-PCR of leaf senescence related genes.**

| **Gene name** | **Primer sequence (5’-3’)** |
| --- | --- |
| actin | ATCCTCCGTCTTGACCTTG |
|  | TGTCCGTCAGGCAACTCAT |
| JZ110587 | GCCTTTGTCGGACTCCTCTGTA |
|  | CTTCGCTGAAAATCTCGTTGC |
| JZ112406 | CACATACTCCCAAGCCCACA |
|  | TGAAAGGAACCAGATCACCCA |
| JZ116048 | AACCCCACTTGCAGCTTTC |
|  | TATTGACCGGACCAACACG |
| JZ116679 | GATACCGTTCCTCACTCTACC |
|  | CTCTCCAATCTCCTTTCCCTAC |
| JZ117638 | CTGGAGCAGCACATAAAGAACC |
|  | ACACCCTCGCGGAGACTAAA |
| JZ117819 | GGTGTCGGTCAACATCAAAT |
|  | CTCCAATGGCAGCAGGTAAT |
| JZ117912 | TACTCGGGTGCTTTGGATTG |
|  | CCTTGAAATTGTGGGGATGA |
| JZ118529 | CACCAAAAGCAGGAACAACAAA |
|  | AGATACAGCCGAACCCTCACTC |
| JZ119002 | AAACCGAATCAGGCAAACAC |
|  | CTCCCTATTGAACCCACCACT |
| Contig1171 | TCAACAAAGAAGGCGAAGGA |
|  | CGTTTAACAGAAGGTCGGAGAT |
| Contig708 | TCAACAGCAAGACATTGGGTTC |
|  | CAGTTCGGCCAGCTTTTAGT |
| JZ112513 | GTTGGACAGTTGTCACCCTGAT |
|  | CAAGATGGGCTGCTATGGAAT |
| JZ111255 | AAACAACCCAAATCCACAGC |
|  | TACGGACTTGAACCACTCTTCTC |
| JZ112420 | TGACAAGTCCCGATTTTGAGG |
|  | ACAGCTTTACAGGCACCACTG |
| Contig1167 | CAGGGAAGACTTACATTCG |
|  | TTTGGCTCCATATCCTTTA |
| JZ112479 | GGTTGAGAATCATCCGAAGCA |
|  | GGACGAAGGAACATAATCGACAG |
| JZ118495 | TTGGCAATGGGGATGTTTC  AATGTCCGAGCGGCTGTTT |
| JZ110276 | ATGGGGCAGAATATGGGATT |
|  | CTTTTGCTGTTGGCACTGTAAC |
| JZ118548 | ATGAAGCAGTGATACAGGC |
|  | CCAGGTACAGCAGCAGAAA |
| GhYLS5 | CTGCTTTGATAATCTGTGGCGAC |
|  | GGCAGTGAAGCATTTTTGACCAG |
| GhYLS8 | TAGACATAACGGAAGTACCTGA |
|  | TTTGTCCTTGAGAGCCCAGTTA |
| GhYLS9 | AGGAGAATCGGAGTACACTATG |
|  | CAGATGCTCGCCCTTGAATAC |
